# Comparison of High-Level Microarray Analysis Methods in the Context of Result Consistency

**DOI:** 10.1371/journal.pone.0128845

**Published:** 2015-06-09

**Authors:** Kornel Chrominski, Magdalena Tkacz

**Affiliations:** University of Silesia, Institute of Computer Science, Division of Information Systems, Sosnowiec, Poland; Queen's University Belfast, UNITED KINGDOM

## Abstract

**Motivation:**

When we were asked for help with high-level microarray data analysis (on Affymetrix HGU-133A microarray), we faced the problem of selecting an appropriate method. We wanted to select a method that would yield "the best result" (detected as many "really" differentially expressed genes (DEGs) as possible, without false positives and false negatives). However, life scientists could not help us – they use their "favorite" method without special argumentation. We also did not find any norm or recommendation. Therefore, we decided to examine it for our own purpose. We considered whether the results obtained using different methods of high-level microarray data analyses – Significant Analysis of Microarrays, Rank Products, Bland-Altman, Mann-Whitney test, T test and the Linear Models for Microarray Data – would be in agreement. Initially, we conducted a comparative analysis of the results on eight real data sets from microarray experiments (from the Array Express database). The results were surprising. On the same array set, the set of DEGs by different methods were significantly different. We also applied the methods to artificial data sets and determined some measures that allow the preparation of the overall scoring of tested methods for future recommendation.

**Results:**

We found a very low level concordance of results from tested methods on real array sets. The number of common DEGs (detected by all six methods on fixed array sets, checked on eight array sets) ranged from 6 to 433 (22,283 total array readings). Results on artificial data sets were better than those on the real data. However, they were not fully satisfying. We scored tested methods on accuracy, recall, precision, f-measure and Matthews correlation coefficient. Based on the overall scoring, the best methods were SAM and LIMMA. We also found TT to be acceptable. The worst scoring was MW. Based on our study, we recommend: 1. Carefully taking into account the need for study when choosing a method, 2. Making high-level analysis with more than one method and then only taking the genes that are common to all methods (which seems to be reasonable) and 3. Being very careful (while summarizing facts) about sets of differentially expressed genes: different methods discover different sets of DEGs.

## Introduction

Microarrays are used to detect gene expression levels. Using this technology, we can simultaneously detect the expression levels of several thousand genes with one experiment [1]. Microarrays can also be used to determine how a disease or other external factors influence the level of gene expression in cells. To reach an appropriate conclusion, it is very important to analyze data (microarray readings) properly. Currently, many methods are used to detect differentially expressed genes (DEGs) from microarray data. However, there is no standardization and every scientist can select his or her preferred method.

When we were asked for help with processing microarray data, we faced the problem of selecting an appropriate method. We were interested in finding a method that would yield "the best result". We found publications that provided comparisons of methods [2, 3, 4, 5]. However, such works did not answer all of our questions. All of the studies proved that methods are not consistent when taking the obtained results into account. At the same time, they did not provide recommendations, standard or procedure proposals or objective method (algorithm) assessments. We also noted that life scientists do not pay special attention to what method they use to analyze the results of microarray experiments (this is partly due to the use of commercial or ready-to-use software, where the information about which method adopted is described in the technical documentation) [6,7]. Based on this, we decided to determine how consistent the results are when examined by different methods of analysis of gene expressions [8, 9]. We decided to describe these results of our study with method evaluation.

We decided to examine six commonly accepted and widely used methods for detecting DEGs [10,11]. The methods we tested were: Significance Analysis of Microarrays (SAM), Rank Products (RP), Bland-Altman (BA), Mann-Whitney test (MW), T Test (TT), and Linear Models for Microarray Data (LIMMA).

Experiments were conducted using real data from eight microarray experiments (hereafter, Arraysets). We found that the first results were surprisingly divergent. Thus, we decided to test the methods on artificially prepared data sets (hereafter, Datasets) with known outstanding values (hereafter, aDEGs—artificial DEG) to be detected.

## Microarray Experiment and Microarray Data Analysis

**Fig 1:**
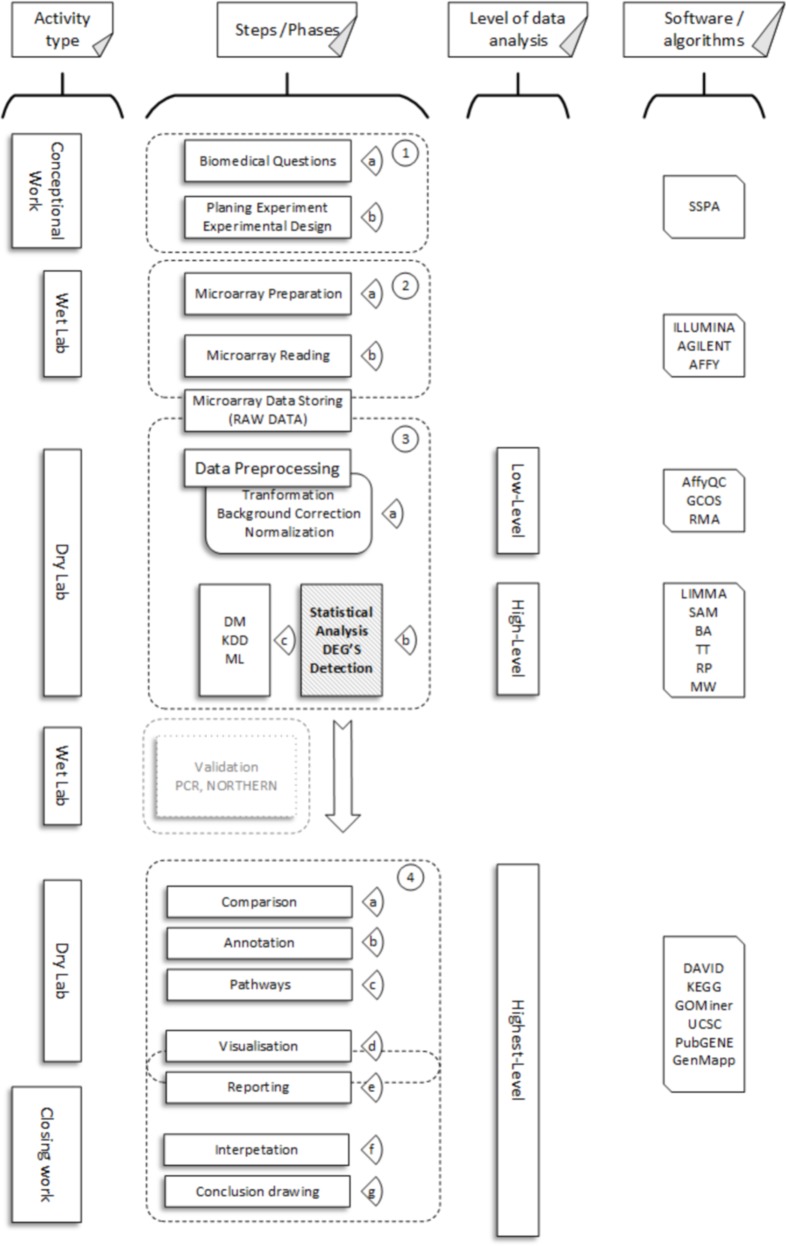
Microarray experiment steps (phases).

To obtain information about the types of microarrays and the principles of their operation, we referred to various sources [12, 13]. Fig 1 presents the steps of microarray experiments (reference to block number is given in brackets).

Aside from the usual steps that are common in most experiments—conception work, laboratory work (wet-lab) and closing work (blocks (1), (2), and (3) (Fig 1, S1 Fig) respectively)—in microarray experiments, three special steps (phases of data analysis) can be specified:

- Low-level data analysis (3a), where the intensity of fluorescence (raw data) is translated into numbers that reflect the fluorescence level for each probesetID for each microarray reading.
- High-level data analysis (3b, 3c), where we exclude probesets without expression changes and select the highest level of data analysis with probesets that undergo expression changes.
- Highest-level data analysis (4), where annotation, pathway analysis, interpretation, reporting, and visualization take place.

In this study, we were only interested in high-level analysis methods, with a special interest in DEGs detection. Because tested method starts with a data table of microarray reading (the table of numbers and probeset identifiers as input data, without additional information), all factors concerning any biological or molecular mechanisms and tissue-specific questions were out of scope in this work. We started our examinations when we saw a normalized table with fluorescence levels for probesets and our goal was to determine which probesets represent genes with transcriptional activity chance (up—and down regulated—for which probeset identifier values changed). In addition, co-expression and pathway analysis was out of scope in our study; this can be done later, based on the results from the high-level analysis. As can be seen (Fig 1), all levels of analyses depended on the results of previous levels. Properly conducted low-level analysis is important for the results of high-level analysis. For highest-level analysis, results of both previous analyses (together with PCR validation of transcriptional activity of certain gene), as well as all biological, molecular mechanisms, and tissue specific issues, are crucial for the final results of experiment.

In short, high-level analysis can be classified as: (3 b) simple methods, which are mainly based on statistics, and (3 c) complex methods, which are based on artificial intelligence and discrete mathematics [14, 15, 16].

### 2.1 Short characteristics of the methods

In the comparisons presented in this paper, we only focused on simple methods. Below, we provide a short characterization of each of the examined methods.

- Significant Analysis of Microarrays (SAM) [17, 18]
  SAM is a statistical method used to determine statistical significance in gene expressions between groups. In terms of mode of action, SAM reassembles a T test. However, SAM uses non-parametric statistics, due to the fact that microarray data are not normally distributed.
- Rank Product (RP) [19, 20, 14]
  RP is a statistical method for detecting gene expression changes. It belongs to non-parametric statistical tests and is based on ranks of fold changes.
- Bland-Altman (BA) [1, 21]
  BA analysis is a statistical method that allows the comparison of two groups of results. In addition to using BA on data from microarray experiments, it is also very popular in medical data analysis of medical data.
- Mann-Whitney (MW) [22]
  MW is a non-parametric test used to test the conformity between two populations. The null hypothesis is that the two populations are identical. It is one of the most popular tests used to check the conformity between groups. One of its usages is to detect gene expression changes in microarray data.
- Test T (TT) [23]
  TT is a statistical test that determines whether two sets differ from one another in a statistically significant way. This test is based on the average and variance of the population. It is one of the simplest and most frequently used statistical tests.
- Linear Models for Microarray Data (LIMMA) [24]
  LIMMA is available as a BioConductor package for analyzing gene expression in microarray data. It uses linear models to analyze microarray data.

We also examined the frequency of using certain methods in scientific papers by searching Google Scholar and PubMed (Table 1).

We searched the name of the method, along with (AND operator) two phrases. In the first search, we used “differentially expressed genes” and in the second, we used "gene expression".

**Table 1:** Frequency of hits: method name along with “differentially expressed genes” and “gene expression” phrases (*Google Scholar*, *PubMed*).

Method name		"Differentially expressed genes"	"Gene expression"
**SAM**	**Significant Analysis of Microarrays**	*1 290;* ***303***	*516;* ***2 287***
	**SAM**	*14 400;* **252**	*190 000*, ***1 746***
**RP**	**Rank Products**	*691;* ***15***	*948;* ***122***
**MW**	**Mann-Whitney**	*7 210;* ***32***	*131 000;* ***511***
**BA**	**Bland-Altman**	*106;* ***2***	*1 930;* ***14***
**TT**	**test t**	*423;* ***1***	*6 420;* ***1 375***
	**t-test**	32 000; ***285***	*1 390 000;* ***1 375***
**LIMMA**	**Linear Models for Microarray Data**	*25;* ***74***	*38;* ***367***
	**LIMMA**	*7 010;* ***77***	*11 600;* ***106***

## Arraysets—Experiments

We compared the results of a high-level analysis for eight microarray (Affymetrix HG-U133A) experiments from ArrayExpress [25, 26]. All readings from this type of microarray contained 22,283 microarray probes (probesets) [12, 27]. The array sets contained microarray readings from a different number of samples. Table 2 presents brief information about the chosen array sets, along with information about the accession number, the number of samples in the array set, and the short characteristics of samples. With the exception of the first array set, all the others included readings from two types of samples: control (from healthy tissue) and tissue from affected tissue. We chose these experiments in order to verify the effectiveness of methods, both in the dependence of the number of samples in the microarray data and the different microarray experiments.

**Table 2:** Arraysets Characteristics.

Arraysets	Accession number	Number of samples	Characteristics
**ArraySet1**	E-GEOD-32072	50	all samples from cancerous tissue (lung)
**ArraySet2**	E-GEOD-14882	16	8—control, 8—patients with MELAS syndrome
**ArraySet3**	E-GEOD-15852	86	43—control, 43—lung cancer tissue
**ArraySet4**	E-MEXP-1690	12	6—control, 6—ganglioglioma
**ArraySet5**	E-GEOD-56899	45	5—control, 40 brain tissue affected by Alzheimer's
**ArraySet6**	E-GEOD-22529	104	82—chemoimmunotherapy patients, 22 from cancer tissue
**ArraySet7**	E-TABM-794	102	50—control, 52—prostate tumours
**ArraySet8**	E-GEOD-11038	72	25—control, 47 tissue with leukemia

The first necessary step was to conduct a low-level data analysis.

**Fig 2:**
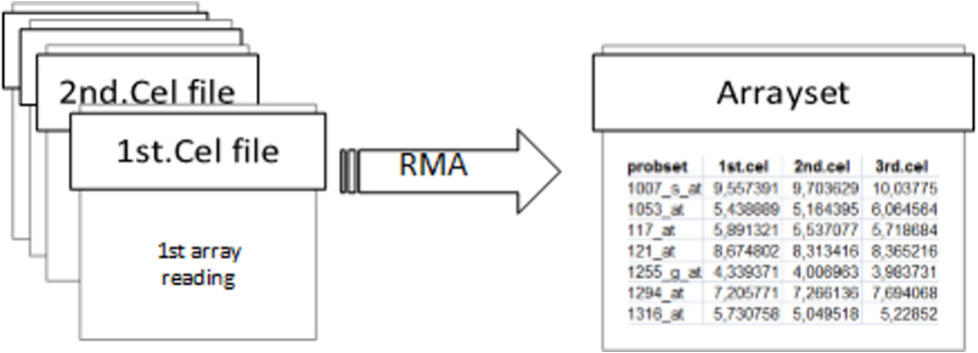
Arraysets preparation process.

In order to perform a low-level analysis, we used standardized Robust MultiChip Average (RMA) [11, 23] method with a Bioconductor [28], which was done for all of the Arraysets. In this study, this type of low-level analysis is not a variable. We present (Fig 2) the process of preparation of Arraysets from the single array experiment data.

For the purpose of conformity verification, we carried out a high-level analysis of array sets. The parameters for each method are presented in Table 3. To perform an analysis of tested methods, we used R packages, or R language built-in functions.

**Table 3:** Parameters that were fixed for each method of high-level analysis (for the purpose of experiments).

Methods	Type of parameter	Value of parameter
**SAM**	fold change	2.00
**RP**	p-value	0.01
**MW**	p-value	0.05
**BA**	p-value	0.02
**TT**	p-value	0.01
**LIMMA**	p-value	0.05

These parameters remained intact throughout all of the testing procedures (both for Arraysets and Datasets).

The number of DEGs detected by different methods is presented in Table 4.

**Table 4:** DEGs detected in Arraysets by different methods (22,283 in all).

	SAM	RP	MW	BA	TT	LIMMA	*common DEGs*
**ArraySet1**	3323	11461	3752	1782	2200	1340	***11***
**ArraySet2**	1043	1446	952	1132	153	952	***6***
**ArraySet3**	4605	4551	2260	1743	1092	2260	***433***
**ArraySet4**	1872	1846	1848	914	320	1848	***91***
**ArraySet5**	11590	2014	840	1476	448	840	***16***
**ArraySet6**	659	3380	992	977	493	992	***95***
**Arrayset7**	2798	3100	4797	2468	2581	2684	***254***
**ArraySet8**	1716	1885	1789	505	1041	633	***122***

From Table 4, it can be seen that we detected a small quantity of common DEGs—from 6 to 433—and the number of DEGs detected by various methods in each Arrayset was different. First, we suspected that the different quantity of detected DEGs was a result of using methods with distinct sensitivity levels. It is for this reason we also examined artificial datasets (see part 4).

**Fig 3:**
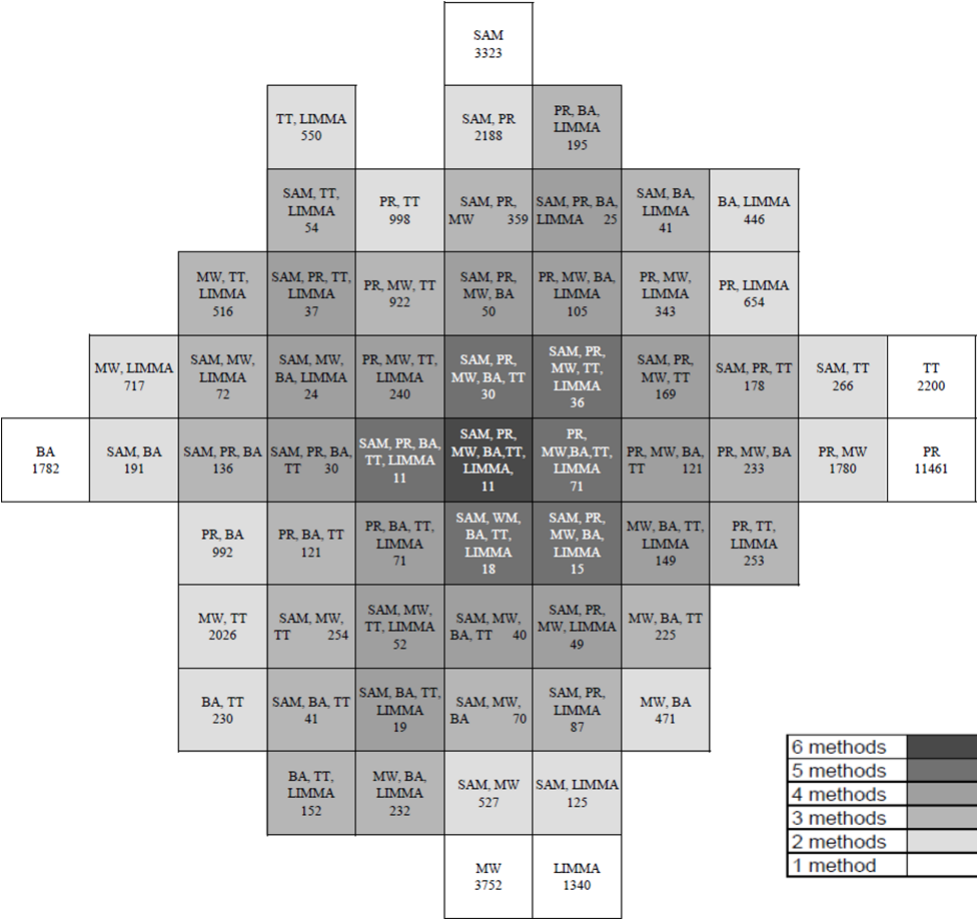
Venn diagram for Arrayset1.

For each Arrayset, we decided to present the results as Venn diagrams. Because the visualization of common parts of more than five sets in the form of Venn diagrams is not trivial, we used the approach presented by [29]. We present the Venn diagram for Arrayset 1 (Fig 3) (Venn diagrams of the remaining Arraysets are presented in Supporting Information files (S1–S7 Figs)). The numbers represent the quantity of detected DEGs. Thus, the diagram also shows the result concordance of methods. The grayscale represents the number of methods, for which the common part has been computed.

On the basis of the presented Venn diagrams, a very low concordance of results between tested methods can be noticed. A large part of DEGs identified by one of the methods was not considered as DEG by other methods. For each Arrayset, the number of DEGs detected by each method can be read in Table 4, and the number of common DEGs detected by methods can be read from the Venn diagrams (Fig 3, S1–S7 Figs).

From the analysis, it is clear the diversified concordance between DEGs detected by certain methods on Arraysets.

**Fig 4:**
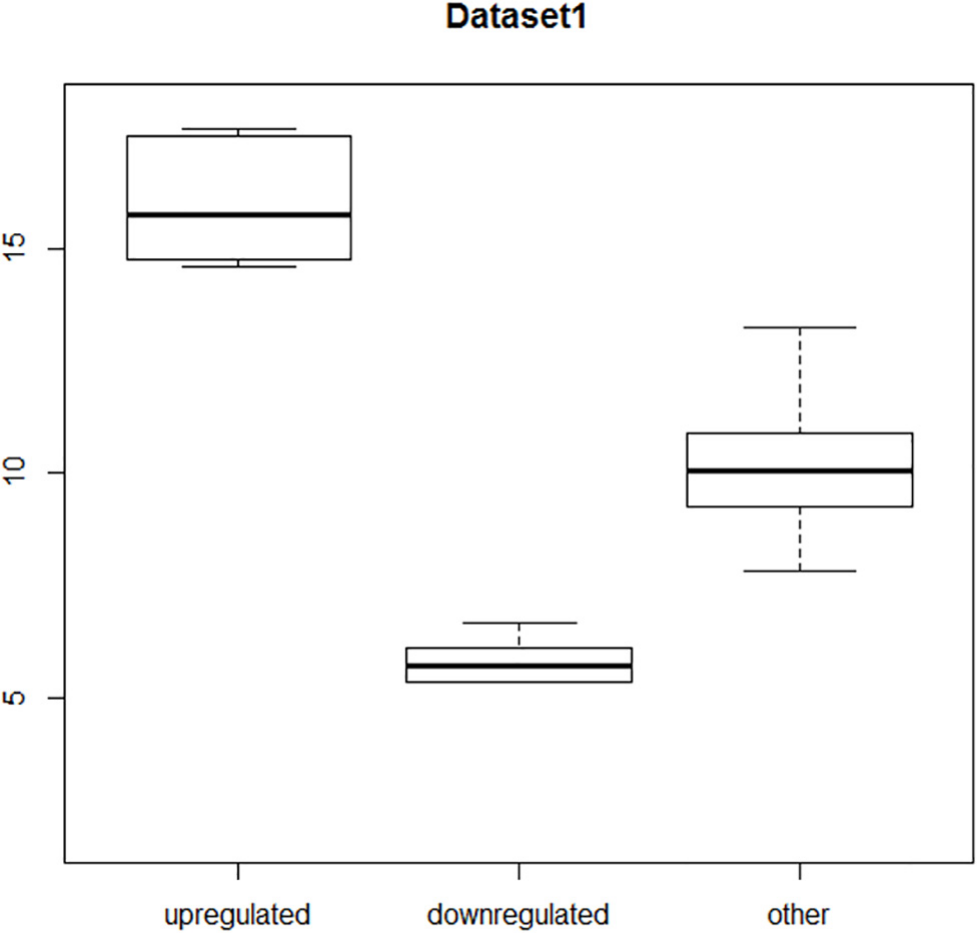
Distribution of values in Dataset1.

Such a low agreement level surprised us. As such, we decided to test the methods on artificially generated data to determine whether the methods themselves (or implementation of algorithms) work properly. We prepared special artificial datasets, with a priori known outstanding in value elements—aDEG (to mimic DEGs). We prepared two datasets with 2,000 elements, generated as normal distribution with mean = 10 and *σ* = 1.3. Next, in the case of Dataset1, 73 elements were changed with values from mean = 15, *σ* = 1.3 (as up regulated aDEGs) to mean = 5, *σ* = 1.3 (as down regulated aDEGs). The range of certain values of exemplary artificial array readings are shown on the boxplot chart in Fig 4 with usual ranges: min value, 1^st^ quartile, median value, 3^rd^ quartile, and max value. The values which "expression" does not change are marked as “other”, up regulated aDEGs are marked as “up regulated”, and down regulated aDEGs are marked as “down regulated”. It can be seen that all ranges are in this case completely separate.

**Fig 5:**
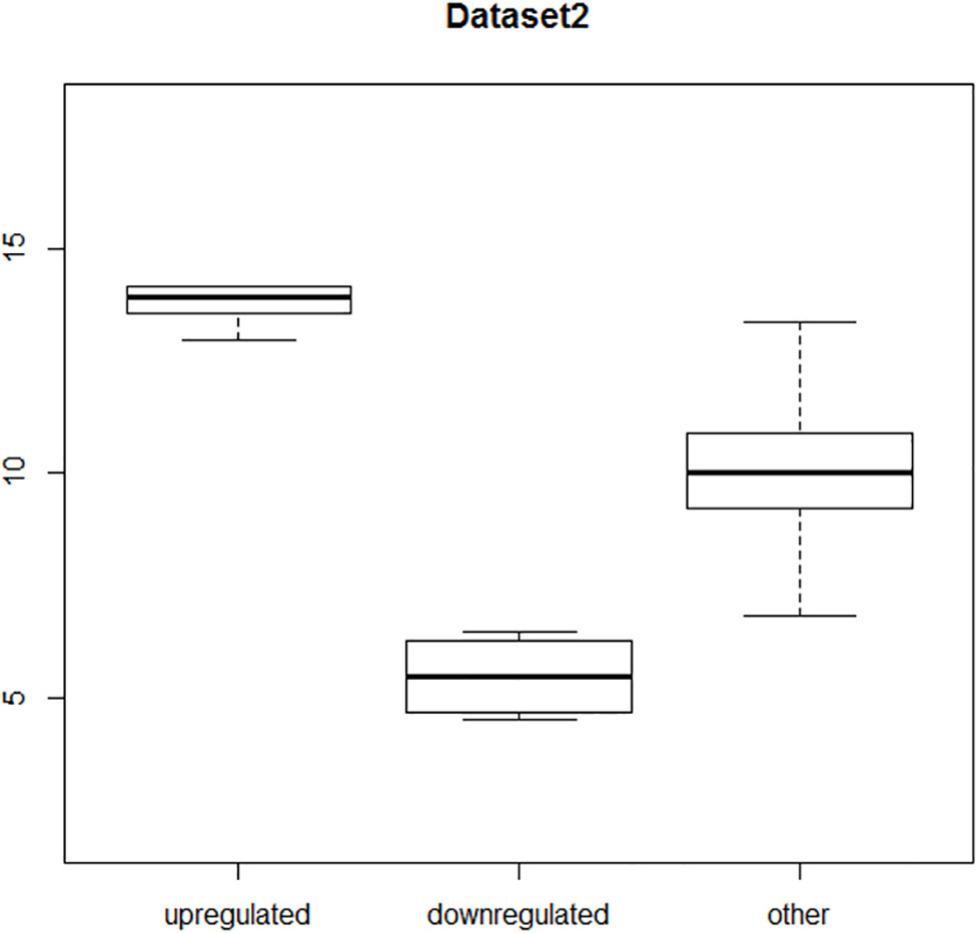
Distribution of values in Dataset2.

Dataset2 had slightly outstanding values—ranging from mean = 13.5, *σ* = 1.3 to mean = 6.5, *σ* = 1.3 respectively (we also wanted to check the sensitivity of each method). Range of certain values for Dataset2 (“other” together with up and down regulated aDEGs) is shown on Fig 5 in a similar way as on Fig 4. In this case, one can see that down regulated are completely separate from “other”, only small amount of up regulated has common range with some maximum values of “other”.

Datasets are uploaded as Supplemental Materials; for more detailed information, refer to adeg_info.txt and ReadMe.txt file in Supplemental Materials. The results of aDEGs detection in both Datasets are presented in the next section.

## Experiments on Datasets

In the case of the Arraysets, it was difficult to assess which method gave the best results, because we did not know which (and how many) values should be detected as DEGs. Thus, we needed a procedure that would allow scoring the methods used for DEGs detection. The problem of detecting DEGs can be regarded as a problem of classifying (grouping) data into three groups: DEGs without change, DEGs up regulated and DEGs down regulated.

### 4.1 Methods Quality Assessment [30, 31]

In the case of the artificially prepared Datasets, we knew what number of aDEGs to detect. Thus, we could make an algorithm assessment. We determined all true positives (*TP*), true negatives (*TN*), false positives (*FP*), and false negatives (*FN*). Therefore, we assessed the quality of methods using measures that were used in computer science during the classification algorithms evaluation. These measures were: accuracy (*acc*), recall (*rec*), precision (*prec*), f-measure (*fm*), and Matthews correlation coefficient (*MCC*). MCC interpretation is similar to ROC/AUC, but it is presented in the form of one number. Furthermore, it is regarded to be more stable when class (groups) sizes may be different. The first used measure was accuracy. Accuracy is defined as:

$$acc=\frac{\left(TP+TN\right)}{\left(TP+TN+FP+FN\right)}$$

Accuracy describes the degree of conformity between values that should be detected and values that algorithms detect. An accuracy value close to 1 means the greater accuracy of the algorithm (it is better). An accuracy value equal to 1 means that the tested algorithm only found the values that should be detected. An accuracy of 0 means that the algorithm has not found any of the values that it should.

The second measure used was recall, also known as true positive rate or sensitivity. Recall is defined as:

$$rec=\frac{TP}{TP+FN}$$

Recall describes how many values are correctly detected by the tested algorithms, in proportion to all of the values that should be detected. Algorithms give the best results when the recall is equal to 1 and the worst results when the recall is 0.

The next measure used was precision. Precision is described as:

$$prec=\frac{TP}{TP+FP}$$

Precision describes how many values are correctly detected by the tested algorithms. As in recall, the best results are given when the precision is equal to 1 and the worst results are given when the precision is 0.

The next metrics was the f-measure, which is described as:

$$fm=\frac{TP+TP}{TP+TP+FP+FN}$$

F-measure is a kind of compromise between recall and precision. When we only use recall or precision, we are not able to decide which algorithm gives better results, detects less "unwanted" values or misses a small amount of that which should be detected. F-measure is usually used for choosing an algorithm with optimal ratio of precision and recall (*fm* = 1 the best, 0 the worst).

The last measure used in the algorithm evaluation was Matthews Correlation Coefficient, which. Matthews correlation coefficient is described as:

$$MCC\;=\frac{TP\cdot TN\;-\;FP\cdot FN}{\sqrt{\left(TP\;+\;FP\;)\cdot (TP\;+\;FN\;)\cdot (TP\;+\;FP\;)\cdot (TN\;+\;FN\;\right)}}$$

Matthews Correlation Coefficient considers values in the range -1 to 1 (-1 the worst, 1 the best). This measure gives information similar to correlation, which inform us to what extent the values that are detected by the tested algorithm are similar to those we expected.

Dataset1 and Dataset2 were examined with the same methods, i.e., SAM, RP, BA, MW, TT, and LIMMA (and the same parameters (see Table 3)).

### 4.2 Dataset1—results

For Dataset1, almost all of the methods detected at least 73 modified values. Furthermore, some recognized additional values. Only the BA method and LIMMA detected less than we expected (72 of 73). The distribution of values in Dataset1 is presented on the boxplot (Fig 4).

A summary of values detected by all tested methods, as well as the measures for method evaluation, is presented in Table 5.

**Table 5:** Number of aDEGs detected and assessment parameters used for each method in Dataset1 (in bold—the best, in *italics*—the worst).

	aDEGs detected (of all 73)					
	SAM	RP	BA	MW	TT	LIMMA
Number of detected values	74	84	76	138	81	72
True positives	73	73	72	73	73	72
True negatives	1926	1916	1923	1862	1919	1927
False positives	1	11	4	65	8	0
False negatives	0	0	1	0	0	1
acc	**0.995**	0.945	0.975	*0*.*675*	0.960	**0.995**
rec	**1**	**1**	*0*.*986*	**1**	**1**	*0*.*986*
prec	0.986	0.869	0.947	*0*.*528*	0.901	**1**
f-measure	**0.993**	0.929	0.966	*0*.*691*	0.948	**0.993**
MCC	**0.989**	0.890	0.947	*0*.*508*	0.918	**0.989**

SAM and LIMMA had the best values in the case of four (for all five) measures. SAM had the highest values for acc, rec, fm, and MCC. LIMMA had the highest values for acc, prec, fm, and MCC. Moreover, SAM and LIMMA reached the same values for acc, fm, and MCC. However, they differed in rec and prec. SAM had the maximum value of rec parameter (equal to 1), which means that the algorithm had no false negatives. LIMMA had prec that equaled 1, meaning that it had no false positives. The worst results were obtained in the case of the MW method. Here, in four (of all five) measures, it had the worst values. Its measures had low values, so even maximum values for rec (equal to 1) would not have justified its usage. BA and TT reached relatively high overall scoring for all measures and the RP results were at an acceptable level.

Table 6 presents a summary of aDEGs detected by various methods. This shows which of the additionally detected values were also detected (or not) by other methods. The first column contains the name of the method used and the second column is the number of additionally detected values, which are also detected by other methods. The other columns show whether additional aDEGs were detected (“Yes”, if detected by another method and “No” if not detected by another method). LIMMA detected less aDEGs than expected, with one false negative, and so it was omitted from this table. For example, in the first row, SAM detected one additional aDEG and this was also detected by MW; in the second row, RP detected one additional aDEG, which was also detected by MW and TT, but not SAM and BA.

**Table 6:** Summary of excessed aDEGs by each method.

	Excessed	Recognized as aDEG by other method					
	aDEG	SAM	RP	BA	MW	TT
**SAM**	1	-	No	No	Yes	No
**RP**	1	No	-	No	Yes	Yes
	4	No	-	No	Yes	No
	1	No	-	Yes	Yes	No
	4	No	-	No	No	No
**BA**	1	No	Yes	-	Yes	No
	1	No	No	-	Yes	Yes
	1	No	No	-	Yes	No
	4	No	No	-	No	No
**MW**	1	Yes	No	No	-	No
	1	No	Yes	No	-	Yes
	1	No	Yes	Yes	-	No
	5	No	Yes	No	-	No
	1	No	No	Yes	-	Yes
	1	No	No	Yes	-	No
	7	No	No	No	-	Yes
	49	No	No	No	-	No
**TT**	1	No	Yes	No	Yes	-
	1	No	No	Yes	Yes	-
	6	No	No	No	Yes	-

It appears that the Dataset1 tested methods worked correctly and were able to detect almost all of the values that should be recognized as aDEGs.

Based on the experiment of Dataset1, we can conclude that the best algorithms for use are SAMM and LIMMA (ex-equo), followed by BA and TT, and, eventually, as a last option, RP and BA. MW had the worst scoring and, therefore, we do not recommend it.

### 4.3 Dataset2—results

A similar evaluation procedure was carried out on Dataset2. Also, like Dataset1, the distribution of values in Dataset2 is presented on boxplot.

Table 7 contains a summary of values detected by all of the tested methods, as well as the measures for method evaluation.

**Table 7:** Number of aDEGs detected and assessment parameters by each method in Dataset2 (in bold—the best, in *italics*—the worst).

	aDEGs detected (of 73 all)					
	SAM	RP	BA	MW	TT	LIMMA
Number of detected values	69	98	50	149	85	76
True positives	69	73	46	73	71	73
True negatives	1927	1902	1923	1851	1913	1924
False positives	0	25	4	76	14	3
False negatives	4	0	27	0	2	0
acc	0.980	0.875	0.845	*0*.*620*	0.920	**0.985**
rec	0.945	**1**	*0*.*630*	**1**	0.972	**1**
prec	**1**	0.744	0.920	*0*.*489*	0.835	0.960
f-measure	0.971	0.853	0.747	*0*.*657*	0.898	**0.979**
MCC	0.957	0.773	0.665	*0*.*443*	0.839	**0.968**

The results for Dataset2 differ from those for Dataset1—they were not so sharp. Similarly to the Dataset1 result, the worst overall result had MW. RP and BA reached better but rather intermediate values in overall range. Additionally, BA had the lowest rec measure for all of the methods. On the other hand, RP had the maximum possible rec measure value. In the case of Dataset2, LIMMA reached the best scores for all of the methods. SAM only had slightly worse scoring. Again, they can be regarded as comparable. The third best-scored method was TT. It had worse results than LIMMA and SAM, but better than BA and RP. Table 8 presents the summary of information about aDEGs (which should be similarly read to Table 6). Due to the fact that SAM detected fewer values than expected, it was omitted from this table.

**Table 8:** Summary of excessed aDEGs by each method.

	Excessed aDEG	Recognized as aDEGs by other method					
		SAM	RP	BA	MW	TT	LIMMA
**RP**	1	NO	-	YES	YES	YES	YES
	5	NO	-	NO	YES	YES	NO
	6	NO	-	NO	YES	NO	NO
	13	NO	-	NO	NO	NO	NO
**BA**	1	NO	YES	-	YES	YES	YES
	1	NO	NO	-	YES	YES	NO
	2	NO	NO	-	NO	NO	NO
**MW**	5	NO	YES	NO	-	YES	NO
	1	NO	YES	YES	-	YES	YES
	6	NO	YES	NO	-	NO	NO
	1	NO	NO	YES	-	YES	NO
	7	NO	NO	NO	-	YES	NO
	56	NO	NO	NO	-	NO	NO
**TT**	5	NO	YES	NO	YES	-	NO
	1	NO	YES	YES	YES	-	YES
	1	NO	NO	YES	YES	-	NO
	7	NO	NO	NO	YES	-	NO
**LIMMA**	1	NO	YES	YES	YES	YES	-
	2	NO	NO	NO	NO	NO	-

It is clear that almost all of the methods detected more aDEGs than was expected—the methods gave worse results on a less diversified Dataset.

Based on the results obtained for Dataset2, the best results had LIMMA, followed by SAM and then TT. RP and BA were also acceptable. Similarly to the experiment with Dataset1, MW gave the worst results.

A final summary of the scoring methods is shown in Table 9.

**Table 9:** Overall scoring of methods for the Datasets (one plus equals one point; the more, the better).

	SAM	RP	BA	MW	TT	LIMMA
Dataset1	+ + +	+	+ +	-	+ +	+ + +
Dataset2	+ + +	+	+	-	+ +	+ + +
overall scoring	**6**	2	3	*0*	4	**6**

Based on Table 9, we recommend methods SAM and LIMMA as the best choice and TT and BA as acceptable for high-level analysis.

## Conclusions

The low level of concordant results for the Arraysets was surprising. When conducted in the Datasets, our analysis showed that, in most cases, the methods themselves (as well as their implementation) work properly (except the MW method, which gave unsatisfactory results). All of the evaluation measures used for scoring methods were better when the outstanding values were well “separated” (more diversified, as in Dataset1). Therefore, one possible conclusion is that microarray experiments data were slightly diversified (similar to Dataset2).

In our opinion, such results show the need to recommend how studies based on microarray experiments should be carried out:

- The list of DEGs should be obligatory, published with precise specification concerning the high-level analysis (and software used).
- When selecting an algorithm for high-level analysis, it is important to take into account the measures for each method and choose a variant method based on special needs (best acc, best rec, best MCC, etc.).
- In general, when the list of DEGs is only obtained with the use of one high-level analysis, it should not be regarded as reliable and definitive. One could argue that an official recommendation about high-level analysis should also be carried out. A possible approach is to use a few methods and acknowledge DEGs as only those genes that are within an intersection of sets of DEGs obtained by different methods. Based on the overall method scoring presented (Table 9), we recommend at least LIMMA, SAM, and TT.

The presented results should also be taken into account by authors of reviews (or those who search for DEGs under certain condition in different papers), while compiling results from different publications that describe a set of detected DEGs. It is very important to pay special attention to the methods of high-level analysis. This is because a resultant set of DEGs can vary, depending on the method used.

## Supporting Information

S1 Data Dataset used in this study. (ZIP)

S1 Fig Venn diagram for Arrayset2. (TIF)

S2 Fig Venn diagram for Arrayset3. (TIF)

S3 Fig Venn diagram for Arrayset4. (TIF)

S4 Fig Venn diagram for Arrayset5. (TIF)

S5 Fig Venn diagram for Arrayset6. (TIFF)

S6 Fig Venn diagram for Arrayset7. (TIF)

S7 Fig Venn diagram for Arrayset8. (TIF)
