# Supplementary figures and images for: Comparison of High-Level Microarray Analysis Methods in the Context of Result Consistency
Source: PLoS One. 2015 Jun 9;10(6):e0128845. doi: 10.1371/journal.pone.0128845 (PMC4461299; doi:10.1371/journal.pone.0128845)

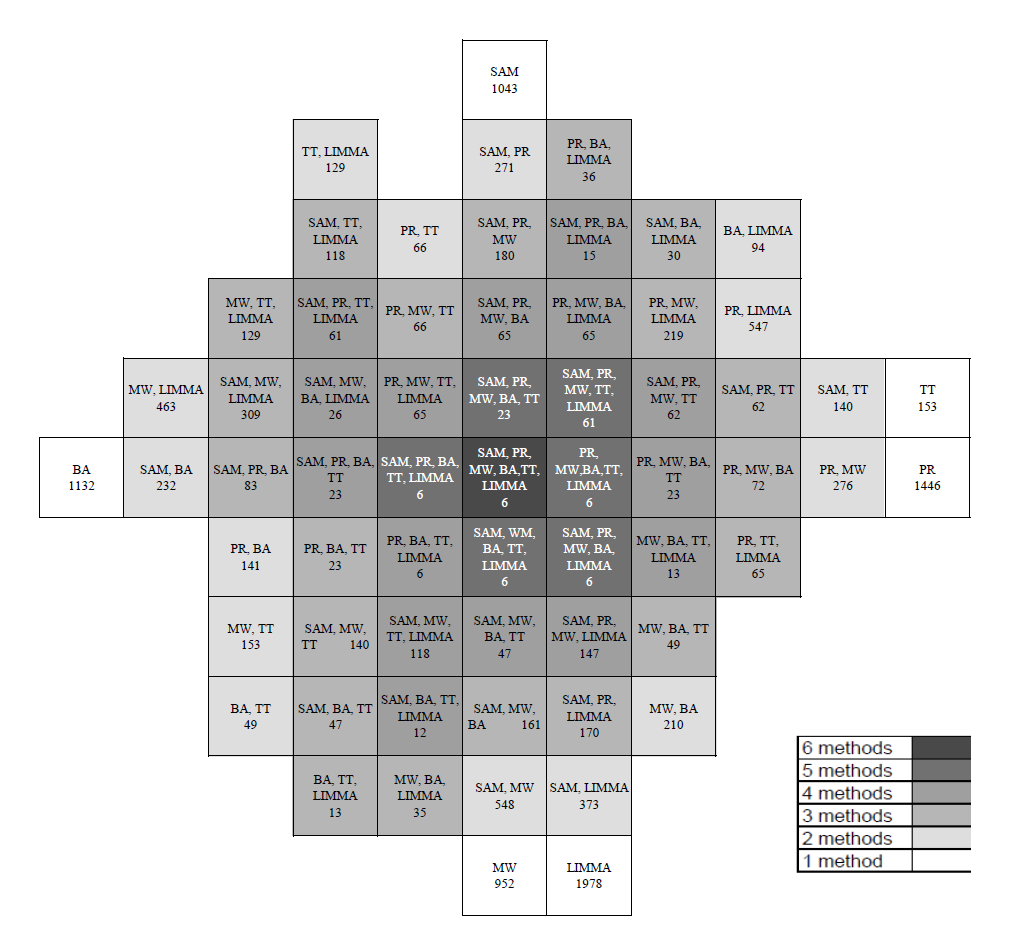

Supplement: S1 Fig — (TIF) [file pone.0128845.s002.tif]

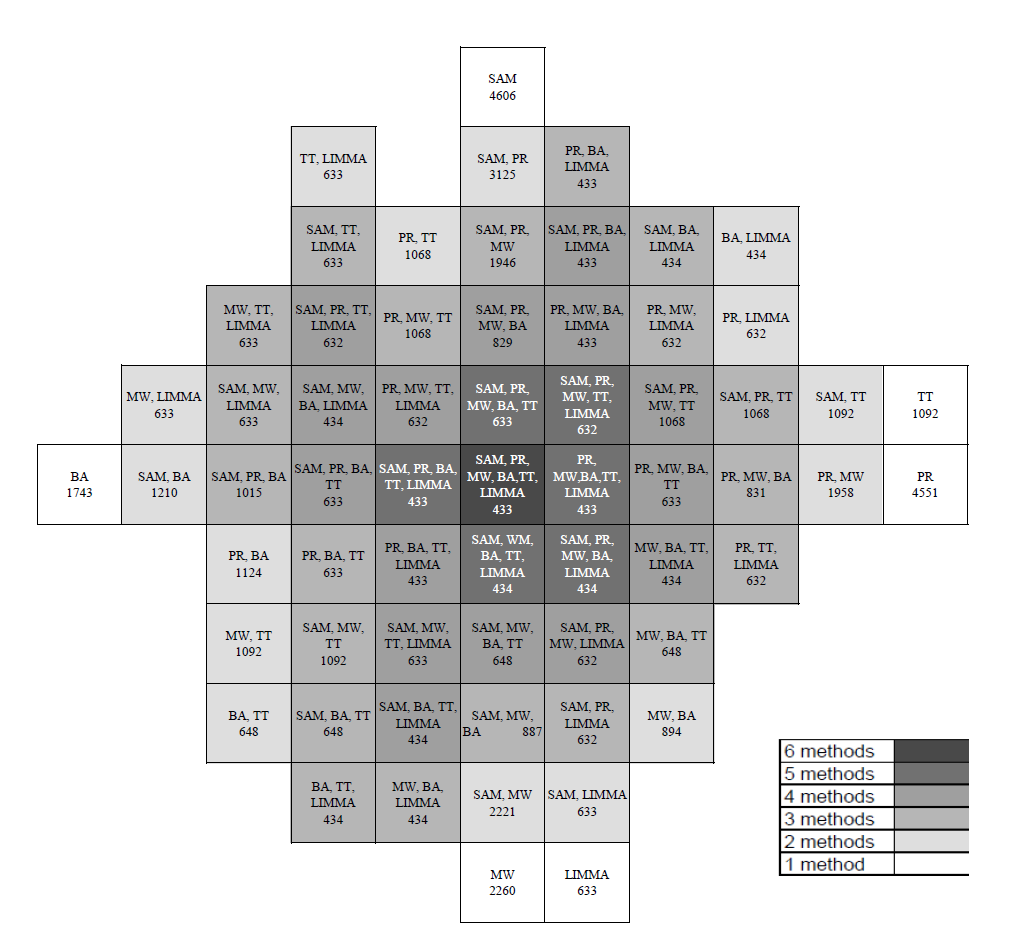

Supplement: S2 Fig — (TIF) [file pone.0128845.s003.tif]

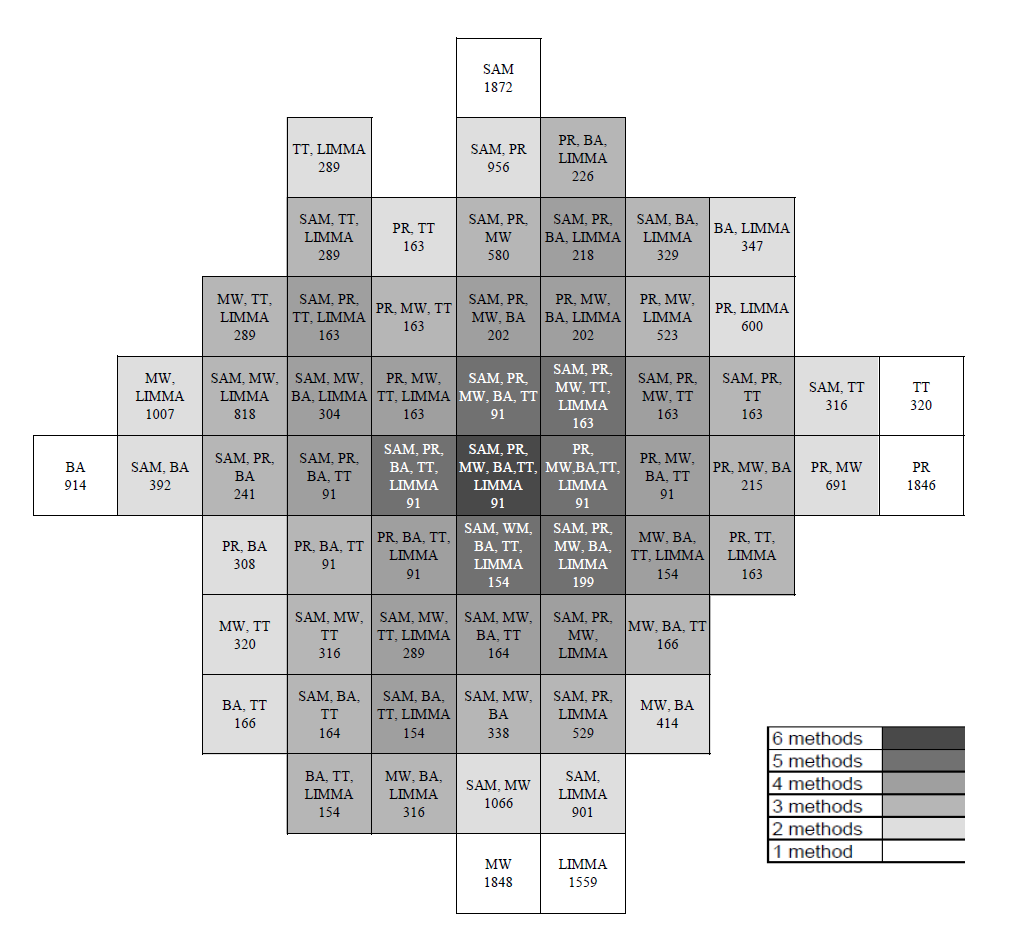

Supplement: S3 Fig — (TIF) [file pone.0128845.s004.tif]

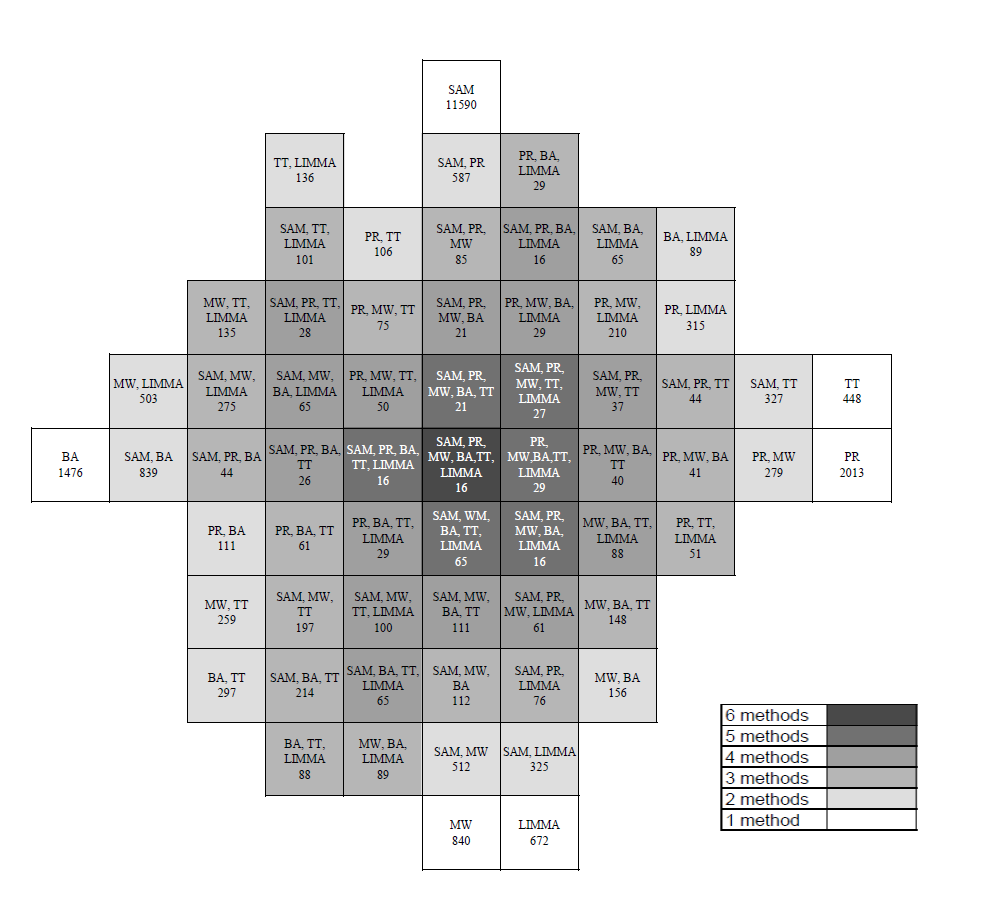

Supplement: S4 Fig — (TIF) [file pone.0128845.s005.tif]

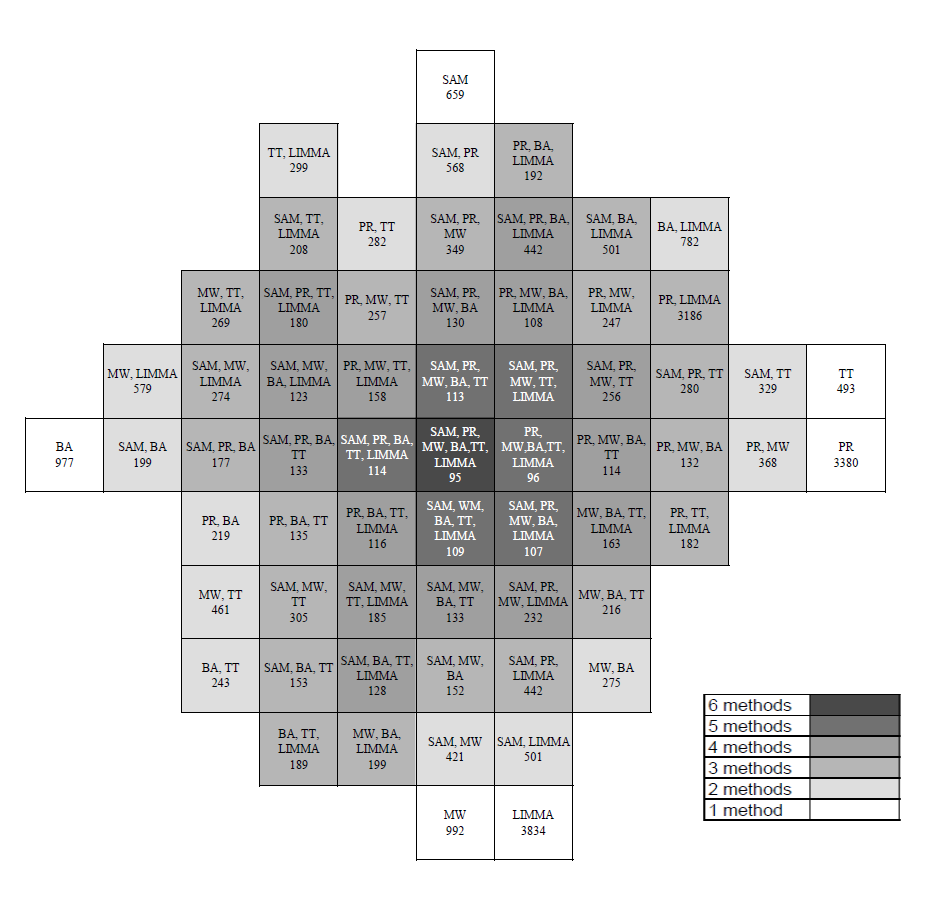

Supplement: S5 Fig — (TIFF) [file pone.0128845.s006.tiff]

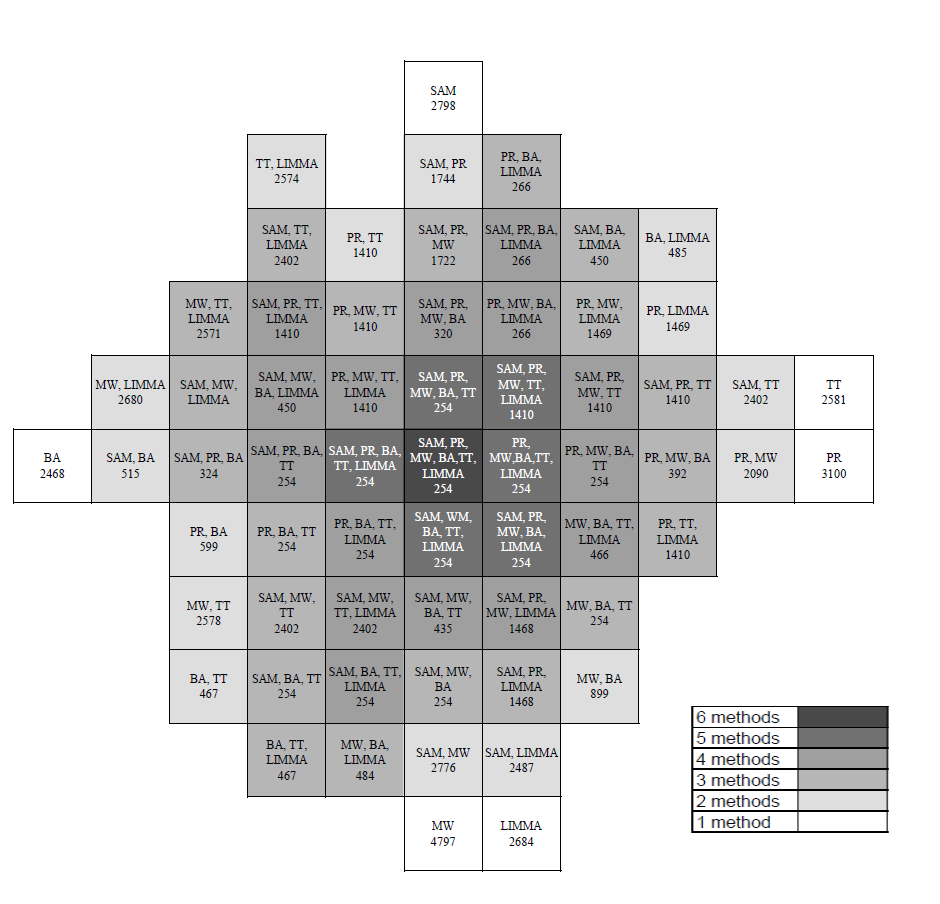

Supplement: S6 Fig — (TIF) [file pone.0128845.s007.tif]

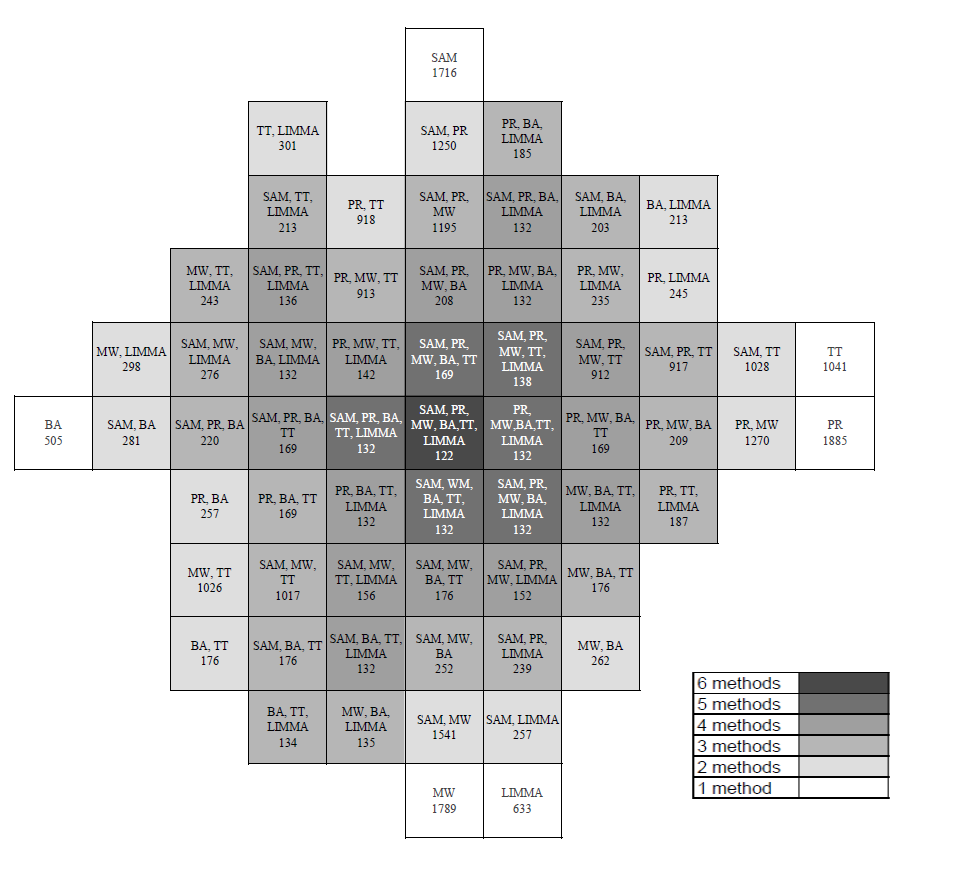

Supplement: S7 Fig — (TIF) [file pone.0128845.s008.tif]
